# The clinical trial activation process: a case study of an Italian public hospital

**DOI:** 10.1186/s13063-024-08059-z

**Published:** 2024-04-05

**Authors:** Carolina Pelazza, Marta Betti, Francesca Marengo, Annalisa Roveta, Antonio Maconi

**Affiliations:** 1 Research Training Innovation Infrastructure, Research and Innovation Department (DAIRI), Azienda Ospedaliero-Universitaria SS. Antonio E Biagio E Cesare Arrigo, Alessandria, Italy; 2 Research Laboratories, Research and Innovation Department (DAIRI), Azienda Ospedaliero-Universitaria SS. Antonio E Biagio E Cesare Arrigo, Alessandria, Italy

**Keywords:** Clinical trials’ activation, Trials’ start-up phase, Trial activation process reorganization, Lean management, Lean thinking, Quality improvement

## Abstract

**Background/aims:**

In order to make the centers more attractive to trial sponsors, in recent years, some research institutions around the world have pursued projects to reorganize the pathway of trial activation, developing new organizational models to improve the activation process and reduce its times.

This study aims at analyzing and reorganizing the start-up phase of trials conducted at the Research and Innovation Department (DAIRI) of the Public Hospital of Alessandria (Italy).

**Methods:**

A project was carried out to reorganize the trial authorization process at DAIRI by involving the three facilities responsible for this pathway: clinical trial center (CTC), ethics committee secretariat (ESC), and administrative coordination (AC).

Lean Thinking methodology was used with the A3 report tool, and the analysis was carried out by monitoring specific key performance indicators, derived from variables representing highlights of the trials’ activation pathway.

The project involved phases of analysis, implementation of identified countermeasures, and monitoring of timelines in eight 4-month periods.

The overall mean and median values of studies activation times were calculated as well as the average times for each facility involved in the process.

**Results:**

In this study, 298 studies both sponsored by research associations and industry with both observational and interventional study design were monitored.

The mean trial activation time was reduced from 218 days before the project to 56 days in the last period monitored.

From the first to the last monitoring period, each facility involved achieved at least a halving of the average time required to carry out its activities in the clinical trials’ activation pathway (CTC: 55 days vs 23, ECS: 25 days vs 8, AC 29 days vs 10).

Average activation time for studies with agreement remains longer than those without agreement (100 days vs. 46).

**Conclusions:**

The reorganization project emphasized the importance of having clinical and administrative staff specifically trained on the trial activation process.

This reorganization led to the development of a standard operating procedure and a tool to monitor the time (KPIs of the process) that can also be implemented in other clinical centers.

**Supplementary Information:**

The online version contains supplementary material available at 10.1186/s13063-024-08059-z.

## Introduction

In the European context, Italy continues to represent a geographical area of great interest for clinical research, an indispensable activity not only for its value in terms of economic investment made in the national territory but also for the opportunity offered to this country to improve clinical practice, grant timely access to therapies to patients, and, more generally, growth.

The scientific excellence of the various Italian trial centers is unfortunately often penalized by the slow process of activating clinical trials, which goes so far as affect Italy’s participation in international studies [1]. This slowness very often is due to the following: (1) a large number of ethics committees and not always adequate; (2) a lack of availability of research infrastructure including dedicated administrative and clinical staff; (3) a complex regulative process [2].

Clinical trials can only start if they have obtained a favorable opinion from the ethics committee. In Italy, interventional pharmacological and medical devices studies also require approval from the competent authorities, Italian Drug Agency (AIFA) and the Ministry of Health, respectively. The request for authorization is based on a complete dossier that includes the study protocol and informed consent for the patient as well as all available information about the experimental products, if any. The structure of the trial dossier is standard and meets the requirements identified in European legislation and transposed into national one, which refer to international scientific standards [3]. State-specific regulations therefore have an impact on the initial phase of trial activation, which therefore makes this phase one of the most complex and costly for sponsors who decide to undertake the initiation of new studies [4, 5].

Currently, through the application of the European Clinical Trials Regulation No. 536/14 [6], this pathway is undergoing a significant reorganization, including a harmonization of clinical trial assessment decisions and administrative processes. This new pathway is fully adopted as of January 2023.

Notwithstanding the quality that a clinical center can demonstrate in the research setting, in terms of qualified personnel and facilities available, the selection of centers conducted by trial sponsors also relies heavily on the timelines required for trial activation. For a clinical center to be selected as a participant in a trial, it must represent an opportunity to provide innovative treatment to its patients. In particular, in trials involving competitive enrollment among participating centers, rapid process for its activation allows a higher rate of recruitment of patients eligible for the experimental treatment.

Difficulties encountered in trial activation have been identified for studies in different therapeutics areas, and pathways have been studied for both industry-sponsored and investigator-sponsored studies [7–9]. In order to make the centers more attractive, in recent years, some research institutes have carried out projects to internally reorganize the pathway for trial activation, identifying the main critical issues and developing new organizational models with a reduction of the time for the start-up phase of trials [10–12]. None of these projects have so far been performed in Italian centers.

This study aims at analyzing and reorganizing the start-up phase of trials conducted at the Research and Innovation Department (DAIRI) of the public hospital “SS Antonio e Biagio e Cesare Arrigo” of Alessandria (AO AL) in Piedmont region (Italy).

## Materials and methods

In the period from November 2019 to August 2022, a project about the reorganization of the trial authorization process was carried out at DAIRI.

### Setting and participants

This process involved different facilities (clinical trial center, ethic committee secretariat, and administrative coordination), everyone with specific roles. The clinical trial center (CTC) was responsible for conducting an assessment of the trial for scientific and economic aspects as well as checking the completeness of the trial dossier provided by the sponsor. Once the assessment has been carried out, the CTC forwards the trial documentation to the secretariat of the Ethic Committee (ECS), which was responsible for including it in the first useful meeting of the Ethic Committee (EC) and producing the minutes of approval. The administrative coordination (AC) was responsible for negotiating the agreement when required and preparing the authorization act for the conduct of the trial at AO AL.

Prior to the start of the project, in September 2019, the trial activation process consisted of these three phases that took place consecutively, and the average time of this process at the hospital was 218 days.

### Lean methodology

The system development methodology refers to the Lean Thinking, a new way to organize processes and activities in different scenarios, including HealthCare, in order to eliminate waste and to optimize resources and to create more value to individuals [13, 14].

Lean Thinking encourages the practice of continuous improvement and is based on the fundamental idea of respect for people. The basis of performance management is the effective use of resources, as measured by quantifying processes and outcomes using key performance indicators (KPIs) [15].

We used the “A3 report” both in the communication process within the team and as a tool for describing, analyzing, and solving the problem (see Additional file 1: Table S1).

The A3 report reflects the results of the whole process, in several different steps:

- Problem description: to clarify the problem and briefly describe it
- Current situation: to describe the current situation in the area where the issue appears and to map the process as it is
- Root cause analysis: to know and fight the root cause of the problem
- Targets/goals: to set goals and step by step to go to the end, also by coming back to previous step and add more details to the initial goals
- Countermeasures: to find and apply solutions
- Implementation: to present an implementation plan of the actions that will be applied
- Results/follow-up: to measure the results and confirm the effect of the applied countermeasures; the step is crucial to set up a continuous improvement

In order to measure and quantify the process improvement, we chose appropriate KPIs (Table 1), and we monitored them by using a graphical dashboard.

**Table 1:** KPIs monitored and explanations of variables used

KPI	Variables used
CTC assessment (days)	Difference between date of completion of documentation and economic and scientific evaluation and date of receipt of trials’ documentation
Pre EC time (days)	Difference between date of ethics committee meeting and date of transmission of trials’ dossier
ECS ethical approval (days)	Difference between date of issuance of approval minutes and date of ethics committee meeting
AC administrative authorization (days)	Difference between date of authorization by the AO AL and date of issuance of approval minutes
AC agreement finalization (days)	Difference between date of last signing of the agreement and date of start of agreement negotiation

### The project

In the first phase of the project, the trial activation process was reviewed as a whole, and all the steps were evaluated to identify initial countermeasures with the aim of streamlining processes and reducing waste. In the second phase of the project, the implementation of the identified countermeasures and the beginning of the new trial activation process was initiated.

After testing these countermeasures, a monitoring period (November 2019–February 2020) of the timeframe of studies activation was carried out, from which additional and more refined countermeasures useful for achieving process standardization emerged. Following the introduction of the last countermeasures, a new phase of monitoring the timing of trial activation started (May 2020–September 2020).

The monitoring period continued even throughout 2021 until August 2022. We defined further six quarters, from September 2020 to August 2022. In each period, we considered all the studies submitted to the local EC.

### Dataset

In order to monitor the timelines related to the process of trials’ activation, a database already in use at the CTC for monitoring active studies was implemented. The variables identified for timing monitoring were based on the activities carried out by the three facilities (CTC, ECS, AC): date of receipt of trials’ documentation, date of completion of documentation and economic and scientific evaluation, date of transmission of trials’ dossier, date of ethics committee meeting, date of issuance of approval minutes, date of start and end of agreement negotiation (if any), date of authorization by the AO AL, date of last signing of the agreement. We considered all trials sponsored by companies or non-profit institutions. For studies sponsored by AO AL, the time of study design and planning was not considered; thus, the time of activation was monitored, since the protocol and attached documents were completed.

The overall mean and median values of studies activation times were calculated as well as the average times for each facility involved in the process. Median activation time values were also calculated by dividing studies with and without an agreement.

## Results

During the eight periods between November 2019 and August 2022, 298 trials were monitored (Table 2).

**Table 2:** Summary of the characteristics of the clinical trials monitored in each quarter

	November 19–February 20	May 20–August 20	September 20–December 20	January 21–April 21	May 21–August 21	September 21–December 21	January 22–April 22	May 22- Aug 22	Tot
Trial design									
Interventional	11	12	14	13	11	14	15	8	98
Observational	15	18	19	24	22	47	34	21	200
Trial nature									
Industrial sponsored	5	11	7	11	8	6	3	5	56
Institutional/investigator initiated	21	19	26	26	25	55	46	24	242
Agreement									
Yes	12	13	13	18	12	16	8	8	100
No	14	17	20	19	21	45	41	21	198
Tot number trial considered	26	30	33	37	33	61	49	29	298
Trial activated	24	30	30	34	33	58	46	27	282
Number and reasons for non-activated trails	2 not completed approval process at local EC		2 not completed approval process at local EC; 1 negative opinion from the national competent authority	2 not completed approval process at local EC; 1 negative opinion from the national competent authority		3 not completed approval process at local EC	3 not completed approval process at local EC	1 not completed approval process at local EC; 1 withdrawn by the sponsor	

Of these, 282 were activated at AO AL, while 13 received conditional or suspensive opinion from the local EC with no response from the sponsor within the defined timeframe, 2 received a negative opinion from the national competent authority (i.e., AIFA), and 1 was withdrawn by the sponsor.

Of the 298 studies, 98 were interventional (e.g., studies on the evaluation of innovative drug therapies or medical devices or the study of diagnostic, surgical, or assistive procedures). The other 200 studies were epidemiological or observational pharmacological or observational medical device studies. Regarding the nature of the studies monitored, 56 were for-profit, while 242 were sponsored by research associations or no-profit organizations. Of all the studies considered, 100 involved the signing of an agreement between the sponsor and the AO AL to conduct the study, not only limited to industry-sponsored studies.

The 298 studies examined in the project were not related to COVID-19 because all trials on this specific topic followed a faster activation process due to national regulations.

**Fig. 1:**
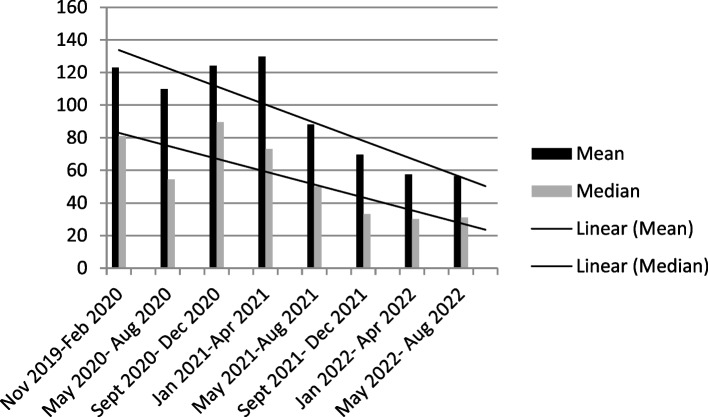
Mean and median activation times of the trials considered in each monitoring period

As a result of the reorganization project, the mean trial activation time was reduced from 123 and 110 days in the first two periods monitored to 56 days in the last period from May to August 2022 (Fig. 1).

Median values decreased from 81 days in period I to 55 days in period II to 31 days in the last two periods monitored.

**Fig. 2:**
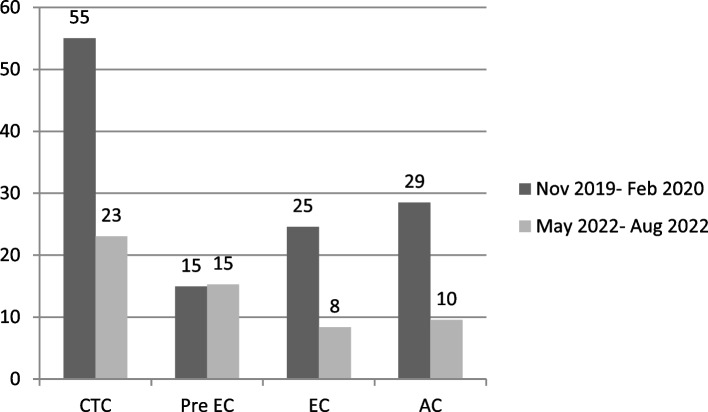
Average times recorded for carrying out the activities of each individual sector in the first and last monitoring period of the trial activation pathway

From the first to the last monitoring period, each facility involved achieved at least a halving of the average time required to carry out its activities in the clinical trials’ activation pathway (Fig. 2).

The average time to carry out CTC activities decreased from 55 to 23 days. The time between the completion of the documentation by the CTC and the submission of the study to the EC cannot be less than 15 days because this is a fixed time stipulated by the study activation procedure. The time required for the ECS to issue approval has decreased from an average of 25 days to 8 days. The time required for approval by the institute, carried out by the AC after EC approval, decreased from an average of 29 days to 10 days.

**Fig. 3:**
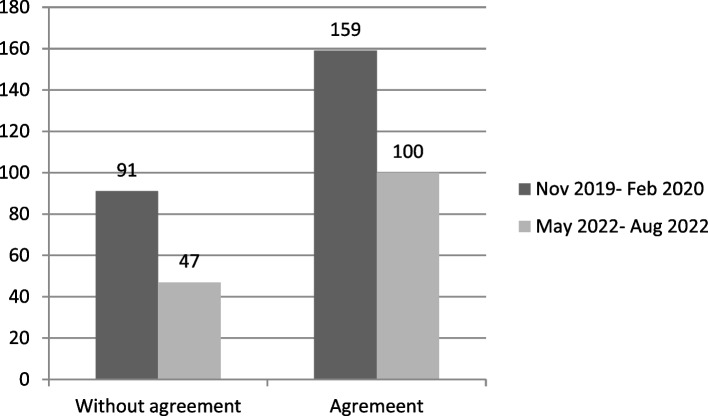
Comparison of the average activation time of trials with and without agreement in the first and last monitoring period

The activation process for studies that do not involve an agreement between sponsor and AO AL has gone from an average time of 91 days in the period from November 2019 to February 2020 to an average of 47 days in the last time frame. The average time frame for activation of studies with agreement has decreased from 159 days in the initial phase to 100 in the last period considered (Fig. 3).

The agreement finalization time (from the beginning of text negotiation to final signature) decreased from 117 to 69 days in the last monitored period.

The current average time of the clinical trial activation process is 56 days and is more influenced by the larger number of studies that do not have agreements and therefore take less time to complete the activation process.

**Fig. 4:**
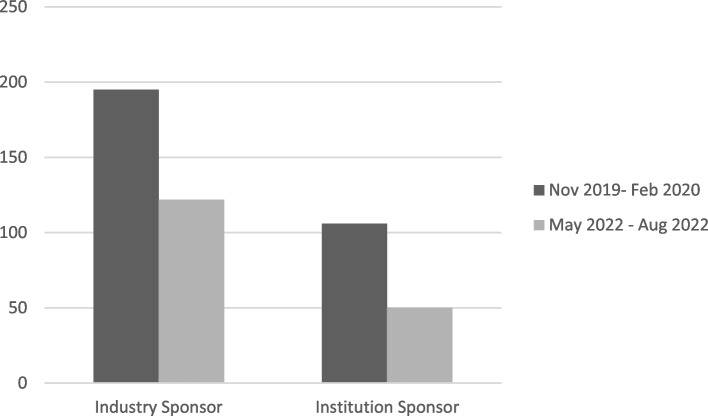
Comparison of the average activation time of industry or institution promoted trials in the first and last monitoring period

Analyzing the data according to the type of sponsor, studies promoted by industry went from an average activation time of 195 days in the first project monitoring period to 122 days in the final one. In comparison, studies promoted by institutions went from an average activation time of 106 days in the period between November 2019 and February 2020 to 50 days in the period between May 2022 and August 2022 (Fig. 4).

## Discussion

At the DAIRI in the Piedmont Region (Italy), a project of the reorganization of the start-up phase led to a reduction from 218 to 56 days of the clinical trial activation process.

During the course of the project, an implementation and reorganization of the staff was also carried out. The turnover of staff involved in this process had a negative impact on the average timelines for the last quarter of 2020 and the first quarter of 2021. This critical issue was agilely overcome thanks to the expertise developed by the working group composed by healthcare (e.g., biologist, research coordinators and data mangers) and administrative staff, which enabled the new staff involved to be trained quickly. This can be observed because the average times monitored in the second quarter of 2021 decreased further compared to those in the second quarter of 2020.

The importance of having trained staff in these specific activities was also observed in another reorganization project [10].

Despite the fact that the project was conducted during the COVID-19 pandemic, we did not identify any factors related to this that may have positively or negatively influenced the average timing of the activation phase of the studies. The studies on this specific topic were not included in the project precisely to avoid a bias because the COVID-19 trials’ activation process was fast-tracked, given the emergency status, according to national legislation.

Active participation in the project to reorganize the clinical trials’ authorization process by all the staff of the three facilities involved enabled them to gain the ability to analyze any criticalities that arise along the way and, through discussion in scheduled meetings, to develop additional countermeasures to facilitate the process.

Among the countermeasures taken that led to the reduced timelines was the identification and training of a group of data managers who check the completeness and assess the suitability of documents before the EC meeting, maintaining contact with the sponsor, principal investigator, clinical research organization (CRO), and ECS. This reduced the number of documentation integration requests from the EC, making the ethics approval phase faster.

In a further move to streamline the EC approval process, ECS compiles a draft opinion before each EC meeting, and once the study is approved, the approval is digitally signed by the EC chair.

The most effective countermeasure applied was to no longer run a linear pathway but to run it in parallel across sectors, as found in similar reorganization projects [10, 11]. According to the current process, the negotiation of the agreement begins in parallel with the evaluation conducted by the CTC, so that once the trial can be forwarded to the EC, the text of the agreement has already been finalized between the AO AL and the promoter (or its delegate). In addition, in order to simplify the agreement negotiation phase, the institution decided to adopt the draft agreement issued by the national authority in charge of drug studies (AIFA) and propose its use to sponsors and CROs.

Finally, in view of the fact that more and more sponsors are requesting digital signature of the agreement, it has been fully adopted, and the director general of the institute has delegated the signing of clinical trial agreements to the head of the AC.

A factor that influences the average timing of the activities carried out by the CTC is the parallel submission of interventional drug trials to the competent authority, coordinating ethics committee, and satellite ethics committee: according to regulations of the local ethics committee, it was not possible to release the approval until it was acquired the favorable approval issued by the coordinating EC. In the case of interventional drug trials, many times the documentation was taken over by the CTC, which carried out the scientific-technical evaluation but then could not proceed to submit the study to the EC.

This delay was eliminated with the full entry into force of the European Clinical Trials Regulation No. 536/14, which, for the activation of this specific type of study, removes the need to submit a trial to several ethics committees in the same country, requiring the approval of only one [6].

The time required to activate a trial is even longer for studies with agreement, because after approval by the institution, there are additional steps, which also depend on the rapidity of the sponsor’s response. This has also been highlighted at other centers that have conducted an analysis related to the management of the trial activation process [9–11].

Comparing the time reduction achieved with that found in other projects [12], it can be considered that the reorganization of the trial activation pathway was successful and that the pathway was fully standardized.

## Conclusions

In conclusion, to the best of our knowledge, this is the first study to analyze and improve the clinical trial activation process in Italy, using the lean thinking methodology.

This study reported an analysis of the clinical trials’ activation pathway in a public hospital of Alessandria in northern Italy. Lean approach allowed a reduction from 218 to 56 days, from documentation intake to authorization act. The hospital includes a Research and Innovation Department characterized by a research infrastructure with facilities as centralized clinical trial center and administrative coordination equipped with dedicated and qualified clinical and administrative.

The reorganization of the complex regulative process led to the development of a standard operating procedure and a tool to monitor the KPIs of the clinical trial activation process that can also be implemented in other clinical centers.

### Supplementary Information

**Supplementary Material 1.**

**Supplementary Material 2.**

## Data Availability

Raw data were generated at DAIRI. Derived data supporting the findings of this study are available from the corresponding author CP on request.

## Abbreviations

DAIRI: Research and Innovation Department
CTC: Clinical trial center
ESC: Ethics committee secretariat
AC: Administrative coordination
KPI: Key performance indicator
AIFA: Italian Drug Agency
AO AL: Public Hospital “SS Antonio e Biagio e Cesare Arrigo” of Alessandria
EC: Ethic committee
CRO: Clinical Research Organization
