# Supplementary material for: The clinical trial activation process: a case study of an Italian public hospital
Source: Trials. 2024 Apr 5;25:240. doi: 10.1186/s13063-024-08059-z (PMC10998293; doi:10.1186/s13063-024-08059-z)
Supplement: Supplementary file 1 — Supplementary Material 1. [file 13063_2024_8059_MOESM1_ESM.docx]

|  | |
| --- | --- |
| *Background* | |
| The activation time for profit and nonprofit clinical trials at Public Hospital "SS Antonio e Biagio e Cesare Arrigo" of Alessandria (AO AL), Italy is long, with an average of 218 days, which is in line with the regional average, but significantly longer than in other Italian regions and especially than in other European countries. | |
| *Current Situation* | |
| 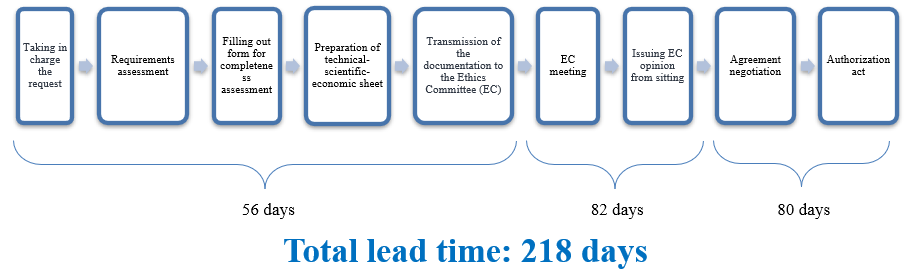 | |
| *Root causes* *analysis* | |
| 1. Trial documentation sent by the Sponsor is often not complete/suitable to be evaluated by the EC 2. The AC manages the negotiation of a multiplicity of trial conventions, sometimes including some that will not be considered at that month's EC meeting 3. EC opinions are completed and sent weeks after the meeting 4. AC revises the agreement after the EC approves the study. The entire negotiation phase between the AO AL and the Sponsor occurs at the end of the process 5. The agreements received by the Sponsors are very different from each other and therefore require burdensome analysis and modifications to be agreed upon with the legal team of the sponsors 6. The authorization act for the conduct of the study takes at least 15 days to be enforceable 7. Many sponsors propose digital signature mode of agreement and the signing of this involves the signature of the General Director of the hospital 8. When the EC issues a conditional favorable or suspensive opinion some sponsors take a long time to provide clarifications and implementations in the documentation | |
| *Target* | |
| Reduce the activation time for clinical trials from an average 218 days to100 days. | |
| *Proposed countermeasures* | |
| 1. A pool of data managers checks for completeness and assesses the suitability of documents prior to the EC meeting, maintaining contact with the Sponsor, Principal Investigator, Clinical Research Organization (CRO), and the ECS. 2. The ECS sends the list of studies that will be evaluated in the next EC meetings to the AC as soon as possible, ensuring priority in finalizing the conventions of those studies. 3. Before each EC meeting, a draft opinion is pre-filled. In addition, the use of digital signature of the opinion by the chair of the EC is introduced. 4. CTC forwards to the AC the draft agreement it receives among the study documents, so that the negotiation is carried out in parallel with the CTC's assessment. 5. The institution decided to adopt the draft agreement issued by the national competent authority on drug studies (AIFA) and to propose its use to Sponsors and CROs. 6. Immediate enforceability of the act is requested in order to proceed quickly with the signing of the agreement between Sponsor and institution. 7. Digital signature mode of agreements is initiated. The Director General delegated the head of the AC to sign agreements for clinical trials. 8. EC stipulated that such responses must be received within 60 days from the date of opinion issues or the practice would be forfeited. This information is included in the minutes of the opinion. |  |
| *Implementation plan* | |
| \| **WHAT** \| **WHO** \| **WHEN** \| \| --- \| --- \| --- \| \| Kick-off meeting \| Coordination Group \| Oct 2019 \| \| Analysis of the current state \| Working Group \| Nov 2019 – Jan 2020 \| \| Meetings to define new operational arrangements \| Working Group \| Jan 2020 – Mar 2020 \| \| Implementation of countermeasures \| Working Group \| Mar 2020 – May 2020 \| \| Testing the new organizational model \| Working Group \| May 2020 – Sept 2020 \| \| Standardization and measurement \| Working Group \| Oct 2020- Aug 2022 \| | |
| *Follow up and expected results* | |
| *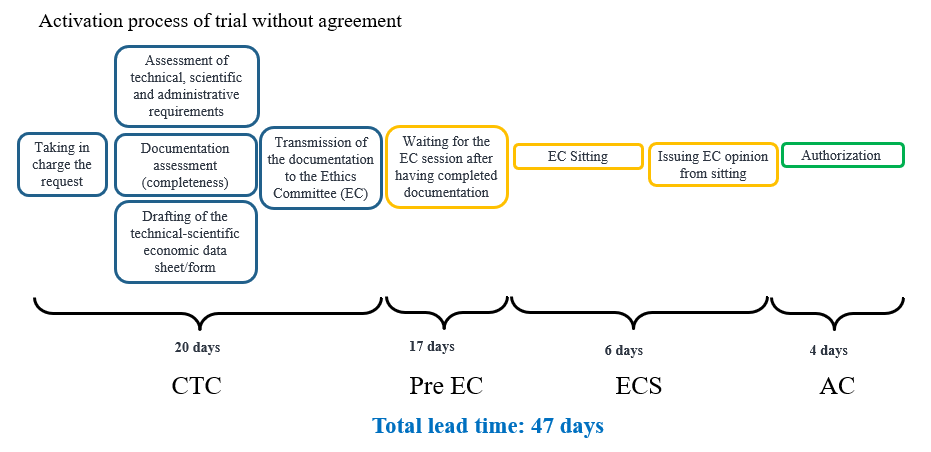 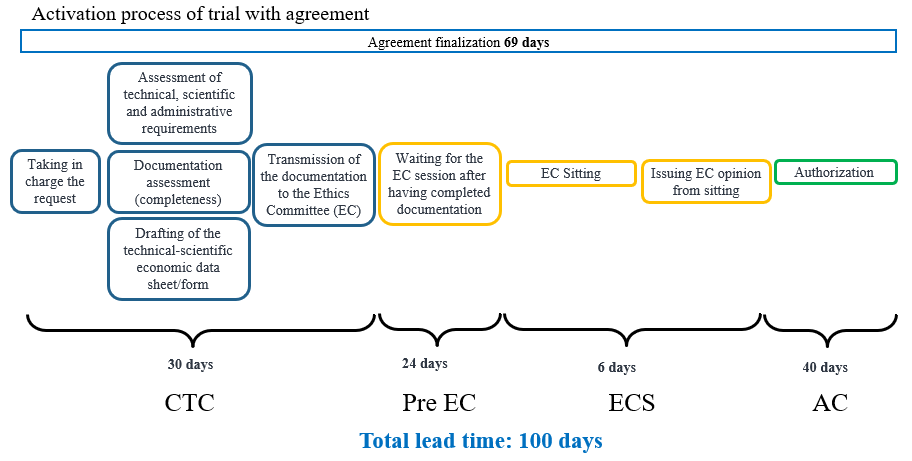* | |

Table 1. A3 report showing background, current situation, root causes analysis, target, proposed countermeasures, implementation plan, follow up and expected results of the project
